# Time-trend of melanoma screening practice by primary care physicians: A meta-regression analysis

**DOI:** 10.1080/03009730802579620

**Published:** 2009-02-04

**Authors:** Antonis Valachis, Davide Mauri, Vassiliki Karampoiki, Nikolaos P Polyzos, Ivan Cortinovis, Georgios Koukourakis, Georgios Zacharias, Apostolos Xilomenos, Maria Tsappi, Giovanni Casazza

**Affiliations:** ^1^PACMeR (Panhellenic Association for Continual Medical Research)AthensGreece; ^2^Dipartimento di Statistica e Biometria, Universita’ Degli Studi di MilanoItaly

**Keywords:** Melanoma, primary care physician, skin cancer screening, skin examination

## Abstract

**Objective:**

To assess whether the proportion of primary care physicians implementing full body skin examination (FBSE) to screen for melanoma changed over time.

**Methods:**

Meta-regression analyses of available data. Data Sources: MEDLINE, ISI, Cochrane Central Register of Controlled Trials.

**Results:**

Fifteen studies surveying 10,336 physicians were included in the analyses. Overall, 15%–82% of them reported to perform FBSE to screen for melanoma. The proportion of physicians using FBSE screening tended to decrease by 1.72% per year (*P* =0.086). Corresponding annual changes in European, North American, and Australian settings were −0.68% (*P* =0.494), −2.02% (*P* =0.044), and +2.59% (*P* =0.010), respectively. Changes were not influenced by national guide-lines.

**Conclusions:**

Considering the increasing incidence of melanoma and other skin malignancies, as well as their relative potential consequences, the FBSE implementation time-trend we retrieved should be considered a worrisome phenomenon.

## Introduction

The incidence rate of melanoma has increased dramatically and persistently throughout the developed and industrialized world over the past 20 years, reaching the proportion of an epidemic disease (1,2). It is estimated that in the United States melanoma will have been diagnosed in about 62,480 persons by the end of 2008, with approximately 8,420 related deaths (2), establishing melanoma as a significant public health issue.

The problem is even more disconcerting considering the limited progress in melanoma therapy over the last decades (3).

Worldwide, overall melanoma mortality is rising, mainly in older men, while rates are decreasing or stabilizing for younger adults (4). Mortality from melanoma is mainly dependent on the thickness of the lesions at diagnosis (5). Indeed, an accurate and timely detection of melanoma is extremely important since early-stage disease is often curable with simple surgical excision; therefore, early detection offers the opportunity to improve survival (6). To date, more than 80% of melanomas are diagnosed at a localized stage, when the cure rate is high (7).

One of the most important early detection strategies is full body skin examination (FBSE) which is painless, rapid, and easy to perform (8,9) and does not require technological skill (4). Full body skin examination can also detect non-melanoma skin cancers (basal cell carcinoma (BCC) and squamous cell carcinoma (SCC)), early detection of which leads to better quality of life and less financial implication for health services (10).

Nevertheless, there is only one randomized controlled trial evaluating the implementation of FBSE among the general population but this was recently disbanded due to lack of governmental funding (11,12). Consequently, since it is unproven (at level I or II of evidence) whether or not skin screening would be effective in reducing mortality from melanoma, guide-lines concerning FBSE lack consensus or are still controversial (13–16) (Table I).

**Table I.** Skin cancer screening recommendations from various organizations.

Organization	Recommendation
American Cancer Society (13)	Age ≥ 20: annual complete skin examination as part of cancer-related check-up
Canadian Task Force on the Periodic Health Examination (14)	There is poor evidence to include or exclude from the periodic health examination of the general population; there is fair evidence for the inclusion of total body skin examination for a very select subgroup of individuals
US Preventive Services Task Force (15)	The evidence is insufficient to recommend for or against routine screening for skin cancer using a total body skin examination for the early detection of cutaneous melanoma, basal cell cancer, or squamous cell skin cancer
American Academy of Dermatology (16)	Annual complete skin examination for all patients

Despite the conflicting evidence, the high curability of melanoma in the early stage and the non-invasive screening procedure with full body skin examination argue for the potential utility of melanoma screening. In this context, since large numbers of the population visit physicians at regular intervals (17), primary care physicians may play an essential role in screening procedures for skin malignancies. Indeed, melanoma patients typically have contact with their physicians in the year before diagnosis (18). Thus, the assistance of primary care physicians may hopefully result in the enhancement of early diagnosis rate.

These considerations led us perform a systematic review of the medical literature in order to evaluate the overall FBSE screening practice among primary care physicians and its trend over time. Taking into account that screening implementation might be influenced by the geographic areas analyzed (Australia versus Europe versus North America), and by national guide-line recommendations, separate meta-regression analyses were performed.

## Methods

### Identification of eligible studies

We searched MEDLINE, ISI Web of Science, and the Cochrane Central Register of Controlled Trials (last search, January 2008) using combinations of terms such as *screening*, *prevention*, *melanoma*, *skin cancer*, *primary care*, *prescription*, and *practice.* We set no language or geographical restriction. We also searched the PACMeR (Panhellenic Association for Continual Medical Research) archives for relevant articles and perused the references of the potentially eligible articles to identify reports that may have been missed by the electronic searches.

### Eligibility criteria

We considered eligible all cross-sectional surveys or controlled trials providing information on the proportion of primary care physicians who reported performing skin examination for screening purposes, with either general population or among high-risk population subgroups. We evaluated all relevant studies, regardless of whether or not the corresponding proportion was a primary outcome. We excluded all qualitative research reports because their sampling methods and stopping rules do not ensure a representative sample and because the thematic coding of the main findings is formulated *post hoc* by the researchers. Physicians with specialties that are usually encountered in non-primary care settings were excluded from the calculations unless it was clearly stated that they were indeed primary care-oriented. We did not consider information pertaining to beliefs or personal views of physicians regarding the role of full body skin examination, as these may be different from actual practice.

### Definitions and outcomes

We considered persons at high risk who were characterized as such in the primary report (e.g. family history, presence of many/atypical moles, fair skin). Screening by continent referred to prevalence of skin cancer screening by physicians in three different regions: Australia/New Zealand, Europe, and North America. Screening by guide-lines referred to prevalence of skin cancer screening by physicians regarding the presence of guide-lines for skin cancer when each study was conducted. Three guide-line categories were used for analysis: 1) national authorities suggest to implement melanoma screening practice among the overall population; 2) national authorities do not advocate implementation of screening practice for melanoma; 3) not assessable (NA) in case national guide-lines were absent or in case national guide-lines from different authorities were conflicting.

We aim to evaluate 1) the proportion of primary care physicians who declare to perform full body skin examination to screen for skin malignancy and its changes over time; 2) the regional distribution of the phenomenon (Australia versus Europe versus North America); and 3) the relative impact of the presence of national guide-lines.

### Data extraction

We extracted information from each eligible study. The data recorded included the first author's name, journal and year of publication, place and country of origin, study design, physician inclusion and exclusion criteria, number of enrolled and analyzed physicians, number of physicians who reported performed or who actually performed full body skin examination for screening, method used to measure study outcomes (standard or telephone interview, questionnaire, patient medical record review or the use of actors paying unannounced visits to the physician), population screened (general population or high-risk subgroup), and definition of high-risk population (if applicable).

In controlled studies that compared the performing rates between a group of physicians who received educational interventions and a control group not exposed to the educational program, only physicians in the control group were considered eligible. Similarly, in interventional studies in which screening attitudes were evaluated before and after an educational intervention, we considered only base-line data (before the educational intervention).

### Analyses

The study was the unit of analyses. For each of the aforementioned outcomes, we calculated whether the proportion of physicians who perform full body skin examination for skin cancer screening changed over time, using random effects meta-regression analyses (19). Meta-regressions are variance-weighted least-square regressions, in which the within-study and between-study variability of the pertinent proportions are accounted for. Summary proportions were estimated using the general inverse variance random effects model (20), which allows for between-study heterogeneity (dissimilarity) and incorporates it into the calculations (21). Heterogeneity in each subgroup was assessed using Fisher's exact test.

Unless otherwise specified, all *P*-values are 2-tailed, and *P*<0.05 indicates formal statistical significance.

## Results

### Eligible studies

**Figure 1.**
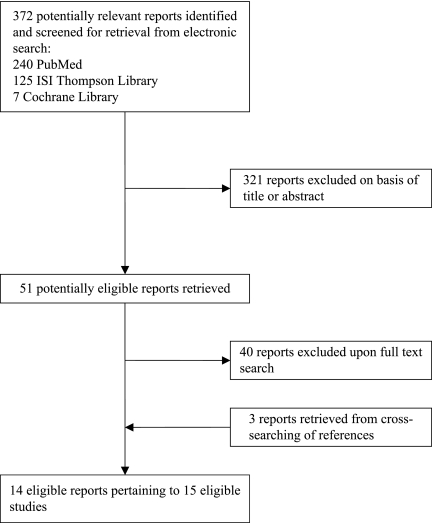
Flow chart of the study selection.

The electronic searches yielded 372 items: 240 from MEDLINE, 125 from ISI Thompson, and 7 from Cochrane Central. Of those, 51 reports were scrutinized in full text. We identified 17 potentially eligible articles. Among those, 14 reports published between 1987 and 2004 were considered eligible (22–35) and pertain to 15 potentially eligible studies. Three reports were considered not eligible since pertaining to beliefs or personal views rather than practices (36–38) (Figure 1). Characteristics of eligible studies are reported in Table II.

**Table II.** Characteristics of eligible studies.

Source, publication year	Enrollment, country	No. analyzed/characteristics of enrolled physicians	Method	Guide-lines	Setting
Resnicow (22), 1987	NA/USA	146 and 129/Randomly selected Society of Teachers of Family Medicine and American Academy of Family Physicians-listed family physicians	Mailed questionnaire	Yes	General population
Harper (23), 1991	1990/New Zealand	210/General physicians in the Canterbury Area Health Board-listed held by the area health board	Mailed questionnaire	Yes	General population
Costanza (24), 1993	1990/USA	488/Primary care physicians in Massachusetts randomly selected from a list provided by the Folio company	Mailed questionnaire	No	General population
Lowe (25), 1994	1991/Australia	46/All GPs from three regional towns in Queensland: Bundaberg, Cairns, Mount Isa	Interview with questionnaire	Yes	General population
Dolan (26), 1995	1994/USA	50/Resident physicians in an academic general internal medicine practice	Mailed questionnaire	No	General and high-risk population
Garcia (27), 1996	NA/France	163/GPs working in Picardy, selected with the assistance of the Union Regionale des Medecins de Picardie	Telephone interview/mailed questionnaire	NA	General population
Sladden (28), 1999	1996/Australia	855/Nationwide random sample of family physicians	Mailed questionnaire	No	General population
Kirsner (29), 1999	NA/USA	191/Random sample of primary care providers, membership enrollment from Dade County, Florida and New Haven, Connecticut	Mailed questionnaire	NA	General and high-risk population
Saraiya (30), 2000	1993–1994/USA	1694/Randomly selected US women physicians from American Medical Association's database	Mailed questionnaire	NA	General population
Altman (31), 2000	1999/USA	1363/Random sample primary care physicians from the Official American Board of Medical Specialists directory of Board-certified medical specialists	Mailed questionnaire	NA	General population
Denise (32), 2003	2002/France	374/General physicians listed in Ordre des Medecines du Haut-Rhin	Mailed questionnaire	NA	General population
Ganry (33), 2004	2003/France	480/General practitioners working in Picardy			
	Mailed questionnaire	NA	General population		
Friedman (34), 2004	NA/USA	247/Random sample from the membership files of the Connecticut state Medical Society	Mailed questionnaire	NA	General population
Geller (35), 2004	2002/USA	380/Randomly selected physicians from American Medical Association's medical marketing services’ database	Mailed questionnaire	NA	General and high-risk population

Overall, 6,816 primary care physicians entered analyses. Sample sizes in the studies ranged from 46 to 1694 analyzed primary care physicians. In all studies, the information about performing FBSE was self-reported.

Three studies were from New Zealand and Australia (23,25,28), three from Europe (France) (27,32,33), and nine from North America (USA). All European studies were from France, while all North American trials were from the USA. Most studies did not include the exact phrasing of the questions used to ask physicians about their skin cancer screening awareness. Similarly, most studies did not evaluate physicians’ beliefs so we could not analyze discrepancies in physician beliefs and attitudes.

### Analyses

**Figure 2.**
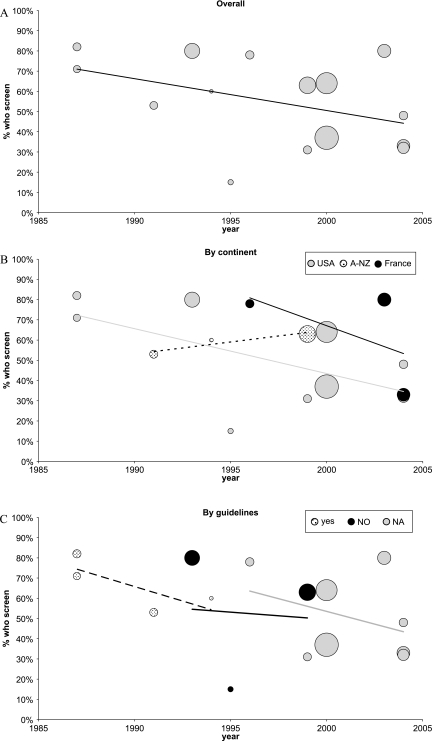
Proportion of primary care physicians who declare to perform full body skin examination over time. A: Overall (all eligible studies included). B: Analysis by continent. C: Analyses by presence of guide-lines. (A-NZ = Australia–New Zealand; Yes = national authorities suggest to implement melanoma screening practice among the overall population; NO = national authorities do not advocate implementation of screening practice for melanoma; NA = not assessable (national guide-lines absent or conflicting)).

*Overall*. 15% to 82% of primary care physicians reported to perform full body skin examination for skin cancer screening purpose. The lowest rate was recorded in a US study (24) when there were guide-lines against skin cancer screening strategies. The highest FBSE rate was recorded in an American study (22) when there were national guide-lines suggesting skin cancer screening in general population. When all studies were considered, the proportion of physicians who performed FBSE seemed to decrease by 1.72% per year, but this was not statistically significant (*P* =0.086; 15 studies with 6,816 analyzed physicians) (Figure 2A).

*Regional distribution*. There is a decreasing trend by 0.68% per year (*P* = 0.494; 3 studies with 1,017 analyzed physicians) in the proportion of French physicians who perform FBSE. The trend became statistically significant when only studies from the USA (*n*=9 studies) were analyzed (annual decrease 2.02%; *P* =0.044). The decreasing trend was reversed when the analysis was related to studies from New Zealand or Australia (*n*=3 studies). We estimated a 2.59% annual increase at FBSE rates in these three studies (*P* =0.010; 1,112 analyzed physicians) (Figure 2B). The proportion of primary care physicians who performed FBSE was more than 50% in all Australian reports.

*Presence of guide-lines*. The presence of national guide-lines was not found to have any statistical significant impact on skin examination performance. We estimated a border-line decreasing tendency of 0.07% per year in the percentage of physicians who perform FBSE (*P* =0.947; 1,393 analyzed physicians) in three studies which were conducted when national guide-lines did not advocate implementation of melanoma screening. This tendency became stronger (annual decrease 0.94%; *P* =0.348) in eight studies with absent or conflicting guide-lines and even stronger, but not statistically significant (annual decrease 1.72%; *P* =0.085), in four studies with guide-lines which suggest skin cancer screening practices in general population (Figure 2C).

*Population at risk*. Only three studies (26,29,35) analyzed the percentage of primary care physicians who performed full body skin examination in high-risk populations. Consequently, due to the paucity of available data, meta-regression analyses were not performed for this outcome. All studies were from the USA and were reporting the proportion of physicians performing FBSE in both general population and high-risk individuals. Overall, we note that the proportion of physicians who report to implement FBSE was increased in the high-risk setting (from 31%, 15%, and 32% in the general population to 52%, 45%, and 59% in high-risk individuals respectively).

## Discussion

Full body skin examination by primary care physicians may be an effective tool in reducing advanced-stage and even mortality rates of melanoma. According to the only population-based randomized controlled trial, the specificity of FBSE for melanoma detection is comparable to that of other established population screening procedures for cancer, including mammography (12). Furthermore, FBSE is able to detect not only melanoma but also many non-melanoma malignant skin lesions (12), and the early detection of these malignancies might result in better quality of life for patients, anticipating major disfigurement, reducing the need for expensive reconstructive surgery, and (to a lesser extent) preventing mortality.

Nonetheless, in the primary care setting the frequency of skin cancer examination rates are low in various reports (26,29,35) and remarkably lower than screening for breast, cervical, and colorectal cancers (31,33,39).

We observed a wide range in the proportion of primary care physicians who perform FBSE for skin cancer screening purposes among eligible studies. Time seemed to reduce the proportion of primary care physicians who perform FBSE. This trend was found to have different geographical patterns, while it is not influenced by the presence/absence of national guide-lines.

Young et al. showed that more than 40% of general practitioners reported that they were not aware of skin cancer screening guide-lines (40). Consequently, the establishment of national guide-lines might not be enough to improve cancer screening participation.

In any case, considering the rising incidence of melanoma, the reduction in melanoma screening procedures is a particularly worrying phenomenon. We can hypothesize that lack of data supporting the value of skin cancer screening, in contribution to adverse effects of screening procedures, might potentially lead to this slow drop. Additionally, while there are no serious risks from FBSE, the examination may be embarrassing to some patients (41). Moreover, a misdiagnosis of melanoma has a serious emotional and financial effect on the patient (42) since it could result in unnecessary treatment. Screening also detects large numbers of benign skin conditions which are very common in the elderly and could lead to additional biopsies and unnecessary or expensive procedures. Thereafter, considering that the Queensland screening trials were disbanded because of lack of governmental funding, there is actually no study able to estimate the balance of potential benefit of screening of skin malignancies. Consequently, in view of the hypothetical screening benefits from indirect evidence (e.g. reduction of mortality), the implementation of randomized controlled trials aiming to evidence real pros and cons of FBSE is essential.

In contrast to French and US practices, in New Zealand and Australia the proportion of primary care physicians who declare to perform full body skin examination for screening purposes is rising. The higher incidence of melanoma in these two regions, compared with the USA and southern European countries (4,43), and the presence of health promotion activities over the past 20 years (44) may be possible explanations for the increasing interest and awareness of skin screening activities (45). Melanoma screening and early detection is considered the most likely cause of the recent statistically significant decreases in mortality observed in Australia (46). The intention to screen is one of the best and most consistent predictors of screening attendance and reattendance (47) and has been shown to be strongly associated with actual screening behavior for breast and colorectal cancer (48,49). Consequently, it is of high importance to organize educational programs for physicians as well as for the general population about skin cancer screening procedures in order to achieve high participation in melanoma screening.

There are some limitations of this study. Firstly, there were only 15 eligible studies. Only three reports were from Australia/New Zealand and three from France. Moreover, there were only three studies with presence of guide-lines respectively against and four studies in favor of skin cancer screening. Therefore, these findings may not be generalized. Secondly, data were derived from cross-sectional studies, which have a limited internal validity and are sensitive to several biases. These surveys have commonly low response rates (50). Furthermore, the vast majority of eligible studies used mailed questionnaires, a method in which incomplete or missing response is likely to be more frequent (51). Overall, 6,816 (65.9%) of 10,336 eligible physicians were analyzed in the eligible studies. This raises further concerns about the generalizability of the results, because non-responders may have systematically different characteristics from those of responders. Finally, the exact question that was used to assess the outcome of interest was not clearly described in most studies. Unfortunately, the effect of different phrasing on our findings cannot be assessed.

Allowing for these caveats, and admitting that such biases may in part affect our study outcomes, the overall results from our comprehensive review are considered to be valid.

### Conclusion

Despite the rising incidence and persistent mortality of melanoma, skin cancer screening rates are shrinking over time. Considering the potential usefulness of skin cancer screening, future efforts should aim firstly to estimate balanced benefits for FBSE screening, and secondly to implement relative evidence-based educational and screening programs. Considering the incidence of melanoma and other skin malignancies, and taking into account the relative potential consequences, the FBSE implementation time-trend we retrieved should be considered a worrying phenomenon.
